# Reported Sildenafil Side Effects in Pediatric Pulmonary Hypertension Patients

**DOI:** 10.3389/fped.2015.00012

**Published:** 2015-03-09

**Authors:** Stephanie L. Siehr, Elisa K. McCarthy, Michelle T. Ogawa, Jeffrey A. Feinstein

**Affiliations:** ^1^Division of Pediatric Cardiology, Department of Pediatrics, Stanford University, Palo Alto, CA, USA

**Keywords:** sildenafil, side effects, pediatrics, pulmonary hypertension, pediatric pulmonary hypertension

## Abstract

**Background:** Sildenafil, a phosphodiestase type 5 inhibitor, was approved in 2005 for the treatment of pulmonary arterial hypertension (PAH) in adults and is commonly used off-label for pediatric patients. Little is known, however, about sildenafil’s side effects in this population.

**Methods:** Single institution, longitudinal survey-based study performed in an outpatient pediatric cardiology clinic. Pediatric patients on sildenafil [alone or in combination with other pulmonary hypertension (PH) therapies] completed questionnaires regarding frequency of vascular, gastrointestinal, neurologic, and hematologic side effects.

**Results:** Between January 2011 and May 2014, 66 pediatric patients with PH on sildenafil filled out 214 surveys, 32 patients (96 surveys) on monotherapy, and 43 patients (118 surveys) on sildenafil plus an endothelin receptor antagonist (ERA) (bosentan or ambrisentan) and/or a prostacyclin (epoprostenol or treprostinil). Overall, 30% of respondents identified at least one side effect. For all patients on sildenafil, incidence of side effects by system was 37% gastrointestinal, 35% vascular, and 22% neurologic. For patients on sildenafil monotherapy, incidence of side effects by system was 24% gastrointestinal, 21% vascular, and 18% neurologic compared to patients on combination therapy who reported an incidence of 48% gastrointestinal, 45% vascular, and 25% neurologic.

**Conclusion:** Incidence of vascular, gastrointestinal, and neurologic side effect in pediatric patients on sildenafil therapy for PAH was 30%. Side effects were more common in patients on combination therapy with an ERA and/or prostacyclin than in patients on sildenafil monotherapy.

## Introduction

Sildenafil, a phosphodiesterase type 5 inhibitor, was approved in 2005 by the Food and Drug Administration (FDA) for the treatment of adults with pulmonary arterial hypertension (PAH). According to the package insert, common side effects include headache, flushing, epistaxis, gastrointestinal distress, and blurred vision (1–3).

As with other therapies for pulmonary hypertension (PH), sildenafil is used off-label for treatment of PH in pediatric patients. The current literature, however, is sparse with respect to side effects in this population. One previous study in the pediatric population reviewed cases reported to the FDA between November 1997 and December 2009 and found 588 pediatric adverse event reports (257 deaths) for sildenafil, bosentan, and epoprostenol. The study was limited, however, by lack of patient specifics provided to the authors by the FDA, and a significant bias in reporting only the most significant events by the care providers to the FDA (4).

Given the reported frequency of adverse events in the pediatric population, and the recent FDA warning (http://www.fda.gov/Drugs/DrugSafety/ucm390876.htm) with regard to the use of sildenafil in pediatric patients, it becomes important to better characterize sildenafil’s side effects and provide clinicians with the data necessary to properly weigh the risks and benefits of its use (5). In this study, we report the incidence of side effects (some previously reported in previous trials and/or product labeling, and others learned through institutional and international community experience) in pediatric patients on sildenafil monotherapy or in combination with other pulmonary vasodilators.

## Materials and Methods

This is a single institution, longitudinal survey-based study performed in an outpatient setting (PH specialty clinic) at a pediatric tertiary hospital. As part of their routine outpatient visits, pediatric patients with PH/pulmonary vascular disease on sildenafil, either monotherapy or in combination with other PH therapies, were given a questionnaire listing common side effects. Patients received questionnaires each time they were seen in clinic and asked to indicate whether the side effect occurred “daily/weekly,” “monthly (or less),” or “never.” Parents filled out paper questionnaires for young children and infants. The Stanford University Institutional Review Board approved the study. Informed consent was obtained from the patient’s parents and assent obtained as appropriate.

The side effects were categorized as gastrointestinal (diarrhea, dyspepsia), vascular (epistaxis, flushing, headache), or neurologic (abnormal vision, hyperactivity, insomnia, myalgia, pyrexia).

### Statistics

Fisher’s exact test was performed to compare the incidence of side effects between patients on sildenafil monotherapy and those on combination therapy. SPSS (IBM SPSS Statistics, Armonk, NY, USA) was used for analysis; statistical significance was set at a *p*-value of <0.05.

## Results

Between January 2011 and May 2014, 66 pediatric patients with PAH on sildenafil filled out 214 surveys, 32 patients (96 surveys) on monotherapy, and 43 patients (118 surveys) on sildenafil plus an endothelin receptor antagonist (ERA) (bosentan or ambrisentan) and/or a prostacyclin (epoprostenol or treprostinil) (Table 1). Therapies were grouped by pharmacologic category as numbers were too small for individual drug comparisons. Patients who started or stopped combination therapy during the course of the study were included, and completed surveys based on their regimen at the time of the clinic visit.

**Table 1:** **Survey “Demographics”**.

Drug	# Patients	# Surveys	PDE-5	ERA		Prostacyclin	
			Sildenafil	Bosentan	Ambrisentan	Epoprostenol	Treprostinil
Dual	10	29	x	x			
	6	9	x		x		
	1	2	x			x	
	11	26	x				x
Triple	1	1	x	x		x	
	6	11	x	x			x
	4	18	x		x	x	
	4	22	x		x		x

*PDE-5, phosphodiestase type 5 inhibitor; ERA, endothelin receptor antagonist*.

The median patient age was 5.7 years (range 0.2–21.6), and each patient/parent completed a median of three surveys over the course of the study (range 1–13). During the course of data collection, one patient had an additional therapy added to sildenafil, eight had adjustments in their combination therapy (e.g., additional or discontinuation of a third medication), and one had combination therapy discontinued, leaving the patient on sildenafil monotherapy.

**Figure 1:**
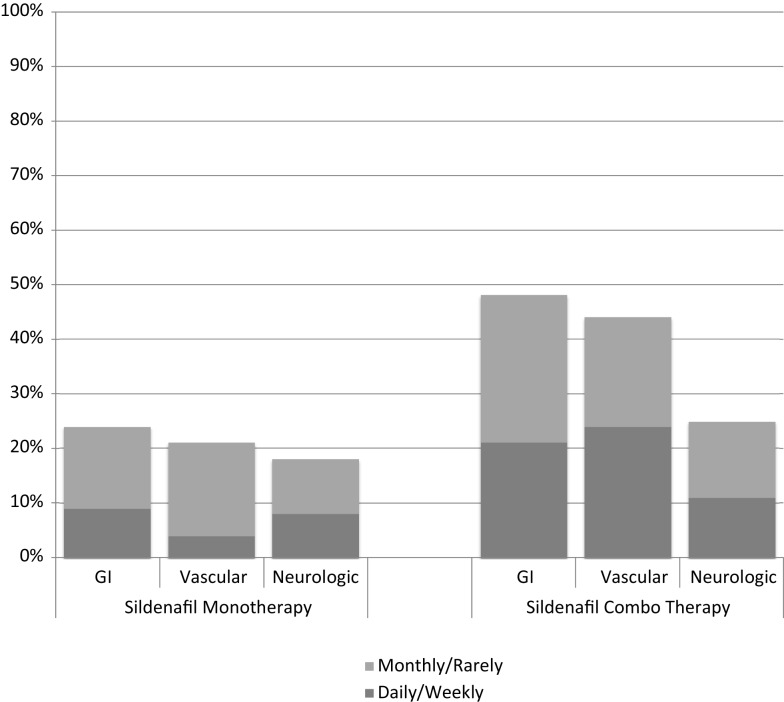
**Side effect frequency on sildenafil monotherapy compared with sildenafil therapy in combination with endothelin receptor antagonists and/or prostacyclin**.

At least one side effect was reported in 30% of the 214 surveys (13% daily or weekly and 17% monthly or less), and at least one side effect on one survey in 94% of the 66 patients. Gastrointestinal side effects were most commonly reported (37% of surveys), followed by vascular (35%) and neurologic (22%) as shown in Table 2. In patients on monotherapy, 24% reported gastrointestinal side effects, 21% vascular, and 18% neurologic. Patients on combination therapy had significantly higher reported side effects: 48% gastrointestinal (*p* < 0.001), 45% vascular (*p* < 0.001), and 25% neurologic (*p* = 0.001). Figure 1 summarizes the side effect frequency by system.

**Table 2:** **Incidence of reported side effects**.

Category	Side effect	Incidence %
Gastrointestinal	Diarrhea	40
	Dyspepsia	35
Vascular	Flushing	41
	Headache	38
	Epistaxis	25
Neurologic	Myalgia	30
	Hyperactivity	26
	Pyrexia	24
	Insomnia	24
	Abnormal vision	9

**Figure 2:**
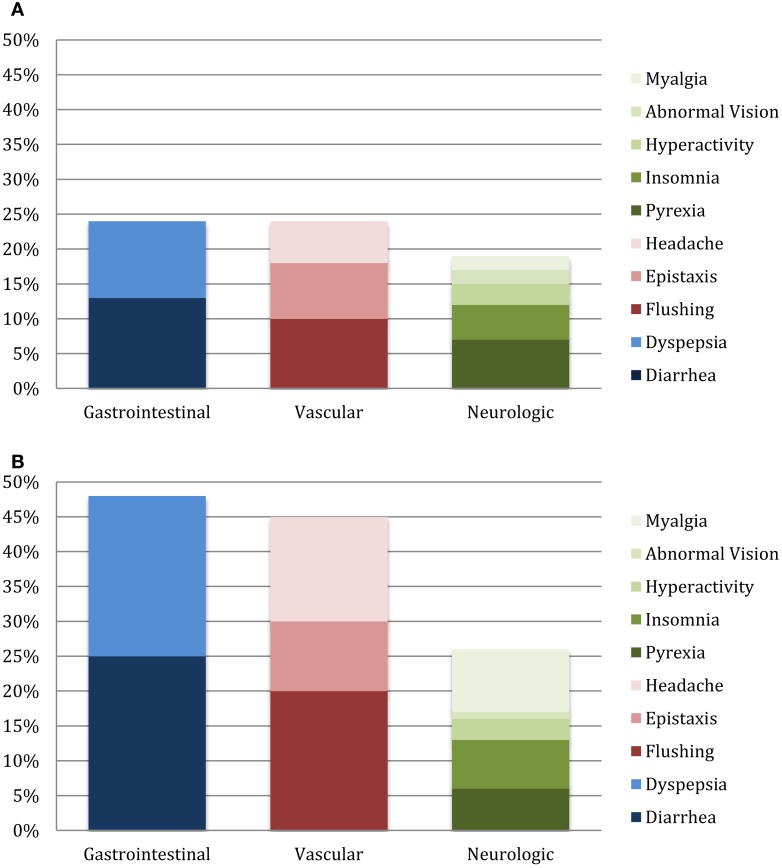
**Side effects on sildenafil monotherapy (A) and combination therapy (B)**.

**Figure 3:**
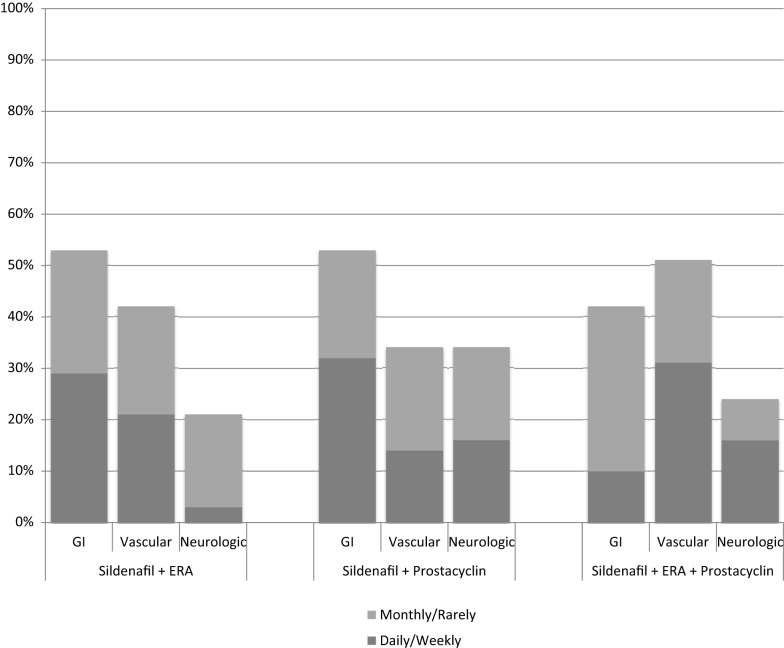
**Side effect frequency for different combinations of sildenafil plus endothelin receptor antagonist (ERA) and/or prostacyclin**.

The most commonly reported side effects on sildenafil monotherapy were diarrhea (26%), hyperactivity (25%), and pyrexia (24%). Dyspepsia and flushing occurred next most commonly in monotherapy patients, each occurring in 22% of patients. On combination therapy, flushing was most commonly reported (57%), followed by diarrhea (51%), headache (48%), and dyspepsia (45%). Monotherapy versus combination therapy side effects are shown in Figure 2. Patients on combination therapy reported gastrointestinal and vascular side effects twice as frequently as patients on monotherapy, while neurologic symptoms were similar except for myalgias (10% monotherapy vs. 42% combination therapy). The frequency of reported side effects by type of combination therapy is illustrated in Figure 3.

## Conclusion

Based on an outpatient questionnaire, 94% of pediatric patients with PH on sildenafil reported at least one side effect on 30% of all surveys, 13% occurring frequently (as defined as daily/weekly). Dosing in all recently initiated patients aligns with current recommendations: 1 mg/kg TID in patients <10 kg, 10 mg TID in patients 10–20 kg, and 20 mg TID in patients >20 kg. Historically, some patients in the 10–20-kg group received 1 mg/kg/dose TID and were included in this study. No patient in this study received more than 1 mg/kg/dose TID (3 mg/kg/day), and no patient was on a dose greater than 20 mg TID.

Side effects were more commonly reported in patients on combination therapy with an ERA and/or prostacyclin than on monotherapy, likely due to a synergistic effect as the side effects reported were the same. The most common side effects both on monotherapy and combination therapy were flushing, diarrhea, dyspepsia, headache, and hyperactivity. Overall, the type of reported side effects was consistent with previous reports in adult patients except for the hyperactivity and insomnia, which have not been previously reported. The incidence of side effects is higher than previously reported.

In the initial Pfizer sponsored randomized clinical trial in adult patients, the most common adverse effects were headache, dyspepsia, flushing, and epistaxis (1). In the longer follow-up analysis of the randomized patients, the adverse effects were consistent with known side effects: headache, dyspepsia, diarrhea, and blurred vision (2). In a Cochrane review of 10 randomized studies of all phosphodiesterase-5 inhibitors approved for treatment of PAH in adults, headache was the most common side effect in a dose dependent fashion, followed by flushing and myalgias (6). Similarly, in combination therapy with epoprostenol, reported side effects were headache, dyspepsia, pain in extremity, and nausea (3).

In an open-label, pilot study of sildenafil use in pediatric patients with PAH that showed improved 6 minute walk test time and hemodynamics, there was a low reported incidence of epistaxis, headache, and flushing (7). The pediatric randomized, controlled trial prompting the FDA warning also reported a variety of side effects, most commonly headache occurring in up to 15% of patients, pyrexia, and vomiting in patients all on sildenafil monotherapy (8, 9). A recent FDA review of reported adverse events in pediatric patients on any therapy for PAH revealed a myriad of adverse events associated with sildenafil therapy not previously reported in sentinel trials, though it is unclear were related to underlying disease progression in patients on monotherapy (4).

Although previous studies have suggested dose-related incidence of the aforementioned side effects, the incidence in this study in patients on a comparatively lower dose was higher than previously reported. A portion of that increase is likely due to the number of patients in this study on combination therapy with an ERA and/or prostacyclin. In addition, the method of self-reporting via a questionnaire in the outpatient setting likely elicits a higher response rate than relying on clinician or patient reported formal adverse events to the FDA. A limitation of this study is that the date of initiation of sildenafil was not included in the questionnaire and, therefore, the time interval from initiation to occurrence of symptoms is not specifically known.

Clinicians prescribing sildenafil for pediatric patients with PAH should inquire about side effects listed here and be prepared to treat appropriately with supportive medications (i.e., anti-diarrheal and acid blocker medications for diarrhea and dyspepsia, respectively). In addition, families should be educated to monitor for these side effects and report them to their provider in a timely fashion to avoid discontinuation of medication if side effects can be appropriately managed.

In conclusion, side effects of sildenafil are common in pediatric PAH patients and more common than previously reported. In addition, side effects occur more commonly when used in combination with other therapies for PAH. Some, such as hyperactivity and insomnia, have not been previously described in adult trials and are important for pediatric clinicians to understand when considering the balance between “cost” and patient benefit.

## Conflict of Interest Statement

The authors declare that the research was conducted in the absence of any commercial or financial relationships that could be construed as a potential conflict of interest.
